# Diagnostic Differentiation Between Unipolar and Bipolar Depression: A Machine Learning Analysis of Demographic and Clinical Features

**DOI:** 10.31083/AP47274

**Published:** 2026-06-30

**Authors:** Lishan Ren, Youdan Wei, Mingjian Cai, Mingfen Song, Kaiyuan Zhang, Zhenghe Yu, Hongjing Mao, Wenjuan Liu

**Affiliations:** ^1^Affiliated Mental Health Centre & Hangzhou Seventh People's Hospital, Zhejiang University School of Medicine, 310013 Hangzhou, Zhejiang, China

**Keywords:** algorithms, bipolar disorder, clinical feature, depressive disorder, machine learning

## Abstract

**Background:**

Differentiating between bipolar depression (BD) and unipolar depression (UD) presents a significant clinical challenge. Identifying the potential clinical features that distinguish between these two disorders is essential for optimizing personalized management strategies for individuals with depression. In this study, we employed machine learning to develop a classification model to distinguish between BD and UD based on demographic and clinical features.

**Methods:**

Patients with either BD or UD were included in this study. Three machine learning classifiers, including logistic regression (LR), random forest (RF), and support vector machine (SVM) were developed and compared using a dual evaluation strategy: (i) nested stratified cross-validation (5-fold outer, 3-fold inner) for unbiased model comparison; and (ii) an independent stratified hold-out split for final validation. In the latter phase, hyperparameters were optimized on the training set via grid search, with performance reported on the test set using bootstrapped 95% confidence intervals. Shapley Additive Explanations (SHAP) analysis was applied to the optimal model to elucidate feature importance.

**Results:**

A total of 449 patients (239 UD and 210 BD) were included. All three models achieved a consistent area under the receiver operating characteristic curve (ROC-AUC) of approximately 0.78, indicating moderate discriminative capacity, with the RF model demonstrating a more balanced error distribution. The top six predictive features were: family history, age, sleep disturbance (Patient Health Questionnaire-9 [PHQ9] item 3), fatigue (PHQ9 item 4), use of sleep medication (Pittsburgh Sleep Quality Index [PSQI] item 6), and suicidal ideation (PHQ9 item 9). The SHAP analysis suggested that younger age, “uncertain/unknown” family history, and the use of sleep medication tended to push predictions toward BD, whereas suicidal ideation, sleep disturbance, and fatigue tended to push predictions toward UD.

**Conclusions:**

Our machine learning approach identified key predictors—including age, family history, and sleep-related symptoms—to differentiate UD from BD in adolescent and young adult patients. Although achieving moderate accuracy, the model may serve as a supportive screening tool to enhance clinical decision-making.

## Main Points

1. Age, family history, and clinical features derived from multiple psychometric scales (specifically patient health questionnaire-9 (PHQ9) and pittsburgh sleep quality index (PSQI)) can distinguish bipolar depression (BD) from unipolar depression (UD) in adolescent and young adult patients.

2. The Random Forest model exhibited the most balanced performance in assessing the classification risk between BD and UD, achieving a receiver operating characteristic area under the curve (ROC-AUC) of 0.78.

3. Shapley Additive Explanations (SHAP) analysis revealed that younger age, use of sleep medication (PSQI item 6), and the “uncertain” family history status (serving as a proxy for clinical ambiguity rather than a biological risk) are the most crucial predictive factors for identifying BD.

## 1. Introduction

Bipolar depression (BD) and unipolar depression (UD) share extensive overlap in clinical presentation during depressive episodes, making accurate differentiation a persistent diagnostic challenge [1]. Depressive episodes are more common than manic or hypomanic episodes in BD [2]. Approximately 85% of BD patients experience a depressive episode as their initial manifestation [3], resulting in nearly 60% being initially misdiagnosed with major depressive disorder (MDD) [4]. Consequently, relying on the history of manic or hypomanic episodes yields low sensitivity for distinguishing BD from MDD, particularly in non-psychiatric settings. Misdiagnosis can lead to the inappropriate use of antidepressants in BD patients, increasing the risk of manic or mixed episodes and exacerbating the severity of the illness [5]. Accurate early differentiation is therefore crucial to ensuring appropriate treatment, reducing healthcare costs, and improving patient quality of life [6]. Predictive factors such as early onset and severe mood symptoms, family history of BD, and emotional dysregulation have been linked to the future development of BD [7, 8].

In recent years, machine learning (ML) has emerged as a promising approach for classifying mood disorders owing to its ability to integrate heterogeneous data sources, including neuroimaging data (structural and functional magnetic resonance imaging [MRI]), clinical rating scales [9], electronic health records, genetic information, and other biomarkers. Studies using multimodal neuroimaging have reported classification accuracies ranging from approximately 70% to 90% for differentiating BD from UD [10, 11]. However, despite these promising results, neuroimaging-based approaches often face limitations such as small sample sizes, high acquisition costs, and limited accessibility, which restrict their routine use in clinical practice. Conversely, studies utilizing purely clinical or combined datasets have achieved competitive accuracies, often exceeding 80% [12]. This suggests that robust clinical markers alone could offer cost-effective and scalable diagnostic tools.

However, translating these findings into routine practice remains challenging due to the nuanced symptom profiles of the two disorders. While BD and UD share a core depressive syndrome, prior research suggests distinct pathophysiological signatures. Specifically, sleep circadian misalignment in BD is often characterized by hypersomnia, delayed sleep phase, and greater rhythm fragmentation, contrasting with the typical insomnia and early awakening seen in UD [13]. Similarly, mechanistic disparities in emotion regulation often manifest as higher severity of comorbid anxiety and somatic burden in BD, likely reflecting heightened inflammatory states or affective instability/impulsivity [14, 15]. In real-world settings, physicians administer a battery of scales (e.g., Pittsburgh Sleep Quality Index, Generalized Anxiety Disorder-7, Patient Health Questionnaire-15) to capture these domains [16, 17]. A recent large-scale cohort study [18] demonstrated that age-dependent developmental trajectories and genetic backgrounds are pivotal in shaping psychiatric outcomes. However, due to the extensive phenotypic overlap, relying solely on aggregate total scores often masks these critical fine-grained distinctions. Consequently, there is a paucity of research dissecting the granular feature importance of these symptoms to identify which specific items beyond broad symptom categories possess the high specificity necessary to disentangle BD from UD.

Based on these findings, the present study aims to develop and evaluate an ML-based classification model that relies solely on routinely collected demographic variables and clinical features derived from multiple psychometric scales. By addressing the cost and accessibility barriers of neuroimaging, this approach offers a resource-efficient screening tool to facilitate early diagnosis in real-world clinical settings.

## 2. Materials and Methods

### 2.1 Study Design and Participants

This retrospective study analyzed clinical data from patients presenting with mood-related symptoms at the Sleep Disorders Clinic of Hangzhou Seventh People’s Hospital of between April 2016 and October 2024. The study was approved by the Ethics Committee of Hangzhou Seventh People’s Hospital (Ethics Approval Number: 2025-005) and adhered to the Declaration of Helsinki. For this retrospective study, the Ethics Committee granted a waiver of informed consent. All patient data were anonymized and stored securely, with access restricted to authorized personnel.

The inclusion criteria were: (1) Age between 12 and 30 years. This range was selected to capture the critical neurodevelopmental period [19] and the peak incidence of mood disorders [20, 21], while also addressing the diagnostic latency common in bipolar disorder [22]; (2) Diagnosis of bipolar I disorder, bipolar II disorder, bipolar disorder not otherwise specified (NOS), major depressive disorder, depressive disorder NOS, or dysthymia. Initial diagnoses were established by psychiatrists using the Structured Clinical Interview for DSM-5 (SCID-5) and retrospectively confirmed via longitudinal follow-up records of at least 2 years to rule out diagnostic conversion; (3) A Patient Health Questionnaire-9 (PHQ9) score ≥5.

The exclusion criteria were: (1) Presence of psychotic symptoms; (2) Comorbidity with other mental disorders, including substance use disorders, personality disorders, or obsessive-compulsive disorder; (3) Presence of severe somatic diseases or unstable medical conditions; (4) History of head injury, organic brain disease, or neurological disorders; (5) Pregnancy or lactation.

### 2.2 Clinical Assessment and Data Acquisition

Demographic and clinical characteristics were retrieved from the “Good Sleep 365” platform (version 4.8.8, Hangzhou Slanhealth Co., Ltd, Hangzhou, Zhejiang, China), where psychometric scales are routinely administered to all patients as part of the standard outpatient assessment. The analysis utilized data recorded at the initial clinical evaluation, irrespective of illness stage, to establish a pre-treatment baseline. The “*Good Sleep 365*” platform is a clinically validated mobile health (mHealth) application that is voluntarily utilized and has been fully integrated into the hospital’s routine clinical workflow. While primarily designed to deliver digital Cognitive Behavioral Therapy for Insomnia (dCBT-I), this app simultaneously functions as an electronic data capture system to support the comprehensive longitudinal management of sleep and mood [23]. Our previous work has demonstrated its clinical efficacy in enhancing treatment adherence and outcomes, as well as its feasibility in real-world settings [24].

To ensure rigorous symptom quantification, we utilized a battery of internationally validated assessment instruments, including the Pittsburgh Sleep Quality Index (PSQI) [25], Generalized Anxiety Disorder-7 (GAD7) [26], Patient Health Questionnaire-9 (PHQ9) [27], Patient Health Questionnaire-15 (PHQ15) [16], and the Epworth Sleepiness Scale (ESS) [28]. These instruments were selected for their robust psychometric properties and demonstrated effectiveness in distinguishing the overlapping phenotypes of somatic symptoms, insomnia, anxiety, and depression commonly observed in BD and UD [16, 17]. The scoring criteria for these instruments are well-documented in the cited references.

### 2.3 Operationalization of Variables

Predictor Variables: To mitigate the risk of multicollinearity and overfitting caused by including redundant variables (i.e., total scores being the sum of item scores), we excluded aggregate total scores from the feature set. Instead, the final model utilized demographic variables (age, family history of mental disorders) and item-level scores from the GAD7, PHQ9, PHQ15, ESS, and PSQI scales. This approach allowed us to explore the differential diagnostic value of specific symptom patterns even when overall severity might be comparable between groups. Our modeling approach integrated demographic predictors alongside clinical scale scores. This strategy aligns with recent large-scale population-based studies, which have demonstrated that incorporating developmental trajectories and genetic proxies is essential for robustly modeling psychiatric outcomes and accounting for biological heterogeneity [18].

Outcome Variable: The outcome variable was the confirmed diagnosis of UD or BD. As detailed in Section 2.1, this diagnosis was ascertained retrospectively via a longitudinal follow-up to minimize the risk of misclassification and diagnostic conversion. All clinical assessments were performed by the same team of psychiatrists and psychologists.

### 2.4 Data Preprocessing

**Fig. 1.**
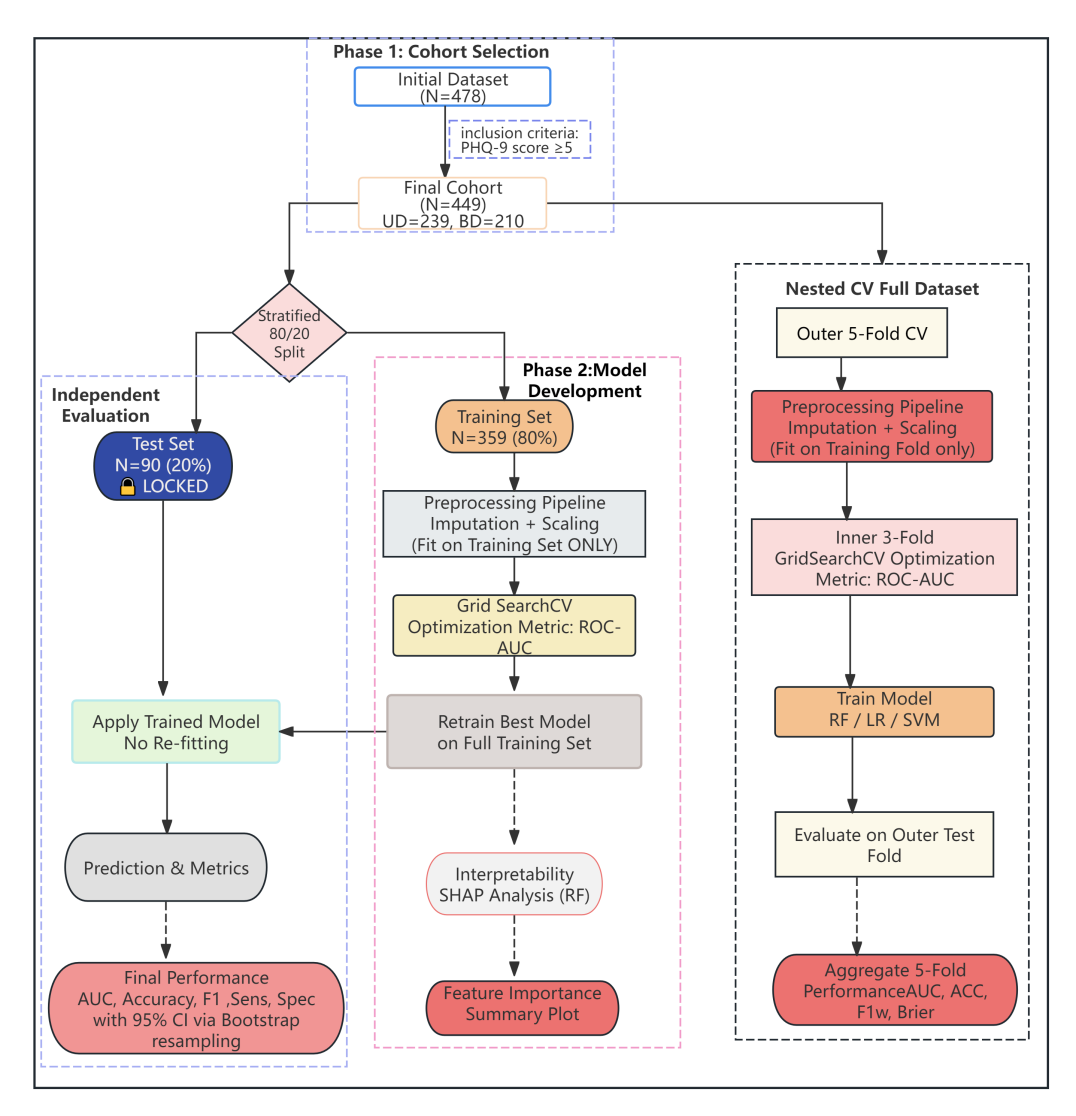
**Schematic illustration of the machine learning workflow**. BD, Bipolar Depression; UD, Unipolar Depression; LR, Logistic Regression; RF, Random Forest; SVM, Support Vector Machine; PHQ9, Patient Health Questionnaire-9; ROC-AUC, area under the receiver operating characteristic curve; SHAP, Shapley Additive Explanations; ACC, Accuracy; CV, Cross-Validation; F1, F1 Score; Sens, Sensitivity; Spec, Specificity; F1w, Weighted F1 Score.

Cohort Selection and Partitioning: Following strict inclusion criteria, eligible records were partitioned into training and test sets at an 80:20 ratio using stratified sampling to preserve class distribution. The training set was used for model construction, while the test set was reserved for independent validation. Fig. 1 was created using WPS Office Software (version 12.1.0.25225, Beijing Kingsoft Office Software, Inc, Beijing, China).

Data Preprocessing and Feature Engineering: A rigorous pipeline was implemented to ensure data quality. First, protocols for missing data were established: mean imputation was designated for continuous variables, and mode (most frequent value) imputation for discrete variables. In this dataset, item-level scale data were complete because the mHealth application enforces mandatory field entry; therefore, imputation would only apply if a missing value were present. Second, to eliminate scale discrepancies, continuous variables underwent standardization. Importantly, standardization parameters (mean and standard deviation) were estimated exclusively from the training set and then applied to the corresponding validation/test set without re-estimation, implemented via a scikit-learn Pipeline (1.3.2, INRIA, Paris, France) to prevent data leakage. Third, categorical variables were numerically encoded; specifically, family history was coded as -1 (Unclear/Unknown), 0 (No), and 1 (Yes). Consequently, the final feature vector comprised 48 predictors: age, family history, and 46 item-level scores from the assessment scales. The outcome variable was binary-encoded, with BD labeled as 1 and UD as 0.

### 2.5 Machine Learning Framework

Classification Algorithms: We conducted a comparative evaluation of three distinct machine learning algorithms: logistic regression (LR) [29], random forest (RF) [30, 31], and Support vector machine (SVM) [32]. Based on comparative performance metrics, the RF model was identified as the optimal algorithm for subsequent analyses. Random Forest is an ensemble learning method based on decision trees that excels in handling complex, high-dimensional datasets [33, 34]. Mechanistically, the algorithm employs bootstrap aggregating (bagging) and random feature selection to construct multiple uncorrelated decision trees. This ensemble approach effectively reduces variance and mitigates the risk of overfitting, thereby enhancing the model’s generalizability to unseen data.

Model training and Performance evaluation: We used a two-stage model evaluation framework to clearly separate algorithm comparison from final performance reporting. First, for model comparison and to reduce optimistic bias during algorithm selection, we conducted a nested stratified cross-validation on the full cohort (outer 5-fold CV for unbiased evaluation; inner 3-fold GridSearchCV for hyperparameter tuning; optimization metric: area under the receiver operating characteristic curve [ROC-AUC]; Fig. 1). Model-comparison results were summarized as the (mean ± SD) across the five outer folds. Second, for final performance reporting with confidence intervals, we performed an independent 80/20 stratified hold-out split. Hyperparameters were tuned on the training set using cross-validation, after which the best-performing model was refit on the full training set and evaluated once on the test set. Predictive performance on the hold-out test set was evaluated using ROC-AUC, sensitivity, specificity, weighted F1 score, and accuracy; 95% confidence intervals were estimated by bootstrapping the hold-out test predictions. To interpret the “black-box” nature of the Random Forest model and identify key predictors, we employed the Shapley Additive Explanations (SHAP) framework [35, 36]. Global feature importance was quantified by calculating the mean absolute SHAP value for each predictor, representing the average magnitude of the feature’s impact on model output.

### 2.6 Statistical Analysis

Statistical analyses regarding demographic and clinical characteristics were performed using SPSS version 20.0 (IBM Corporation, Armonk, NY, United States). Since continuous variables—including age and symptom scales (GAD7, PHQ9, PSQI, ESS, and PHQ15)—exhibited non-normal distributions, they were expressed as medians (Q1(25th percentile), Q3(75th percentile)) and compared using the Mann–Whitney U test. Categorical variables, such as sex, educational level, and family history, were analyzed using the Chi-square test. To quantify the magnitude of differences, Cramér’s V and Cliff’s delta were calculated as effect sizes, respectively. To control for Type I errors, all *p*-values were adjusted for the false discovery rate (FDR) using the Benjamini-Hochberg procedure. Statistical significance was defined as a two-tailed *p*-value < 0.05. Stratified analyses were performed by age and family history, with additional sensitivity analyses conducted for family history. In the machine learning phase, we utilized the Scikit-learn library (Python 3.10.12, Python Software Foundation, Beaverton, Oregon, USA) to implement and compare three distinct classification algorithms: LR, SVM, and RF. This data-driven modeling approach was adopted to align with analytic standards established in large-scale psychiatric studies (Koutsouleris *et al*. [37]), thereby ensuring methodological rigor and reproducibility.

## 3. Results

### 3.1 Demographic and Clinical Characteristics

A total of 449 participants were included in the study, consisting of 239 patients with UD and 210 patients with BD Comparisons of demographic and clinical variables are presented in Table 1. Regarding demographic characteristics, significant differences were observed in age and family history. Patients in the UD group were significantly older (median [Md] = 23 years) compared to the BD group (Md = 19 years; Z = –5.7, *p*FDR <0.001), with a medium effect size of –0.31. Significant differences were also found in family history distributions (χ^2^ = 83.9, *p*FDR <0.001), with a moderate-to-large effect size of 0.43. While the UD group had a higher proportion of participants with a university education or above compared to the BD group (62.8% vs. 48.1%), this difference was not statistically significant after FDR correction (*p* = 0.002, *p*FDR = 0.07). No significant difference was found in sex distribution. Regarding specific clinical symptoms, several items remained significant after FDR correction: PHQ9_3, PHQ9_4, PHQ9_6, and PHQ9_9; PSQI_3; and PHQ15_7 and PHQ15_9 (all *p*FDR < 0.05).

**Table 1.** **Clinical characteristics of UD and BD**.

Variables		UD (239)	BD (210)	χ^2^/Z	Effect size	*p*	*p*FDR
Age (years) Md (Q1, Q3)		23 (18, 27)	19 (16, 23)	–5.7^a^	–0.31	<0.001^*^	<0.001^*^
Sex (female/male)		161/78	156/54	2.6^b^	0.07	0.120	0.30
Educational level (n,%)	Primary and secondary school	89 (37.2%)	109 (51.9%)	9.8^b^	0.16	0.002^*^	0.07
	University and above	150 (62.8%)	101 (48.1%)				
Family history (N/Y/Unclear) (n,%)		117 (49.0%)/64 (26.8%)/58 (24.3%)	49 (23.3%)/20 (9.5%)/141 (67.1%)	83.9^b^	0.43	<0.001^*^	<0.001^*^
GAD7_4 Md (Q1, Q3)		2 (1, 3)	2 (1, 3)	–2.3^a^	–0.12	0.021^*^	0.07
PHQ9 items Md (Q1, Q3)	PHQ9_3	3 (2, 3)	2 (1, 3)	–4.0^a^	–0.20	<0.001^*^	<0.001^*^
	PHQ9_4	2 (2, 3)	2 (1, 3)	–3.7^a^	–0.12	<0.001^*^	0.02^*^
	PHQ9_6	3 (2, 3)	3 (1, 3)	–3.1^a^	–0.15	0.002^*^	0.02^*^
	PHQ9_8	2 (1, 3)	2 (1, 3)	–2.6^a^	–0.12	0.019^*^	0.07
	PHQ9_9	2 (1, 3)	2 (1, 3)	–2.6^a^	–0.14	0.009^*^	0.05
PSQI items Md (Q1, Q3)	PSQI_1	2 (2, 3)	2 (2, 3)	–1.9^a^	–0.10	0.047^*^	0.14
	PSQI_3	2 (0, 3)	2 (0, 3)	–2.9^a^	–0.15	0.004^*^	0.03^*^
	PSQI_4	2 (0, 3)	1 (0, 2)	–2.4^a^	–0.12	0.017^*^	0.07
PHQ15 items Md (Q1, Q3)	PHQ15_7	2 (2, 2)	2 (1, 2)	–3.1^a^	–0.12	0.002^*^	0.02^*^
	PHQ15_9	0 (0, 1)	1 (0, 1)	–2.6^a^	–0.13	0.009^*^	0.05
	PHQ15_10	0 (0, 1)	1 (0, 1)	–1.9^a^	0.10	0.049^*^	0.14
	PHQ15_11	0 (0, 1)	1 (0, 1)	–2.1^a^	0.10	0.038^*^	0.13
	PHQ15_15	0 (0, 1)	0 (0, 1)	–2.4^a^	0.11	0.017^*^	0.07

Md, median; Q1, 25th percentile; Q3, 75th percentile; GAD7, Generalized Anxiety Disorder 7-item scale; GAD7_4, Trouble relaxing; PHQ9_3, Sleep Disturbance; PHQ9_4, Fatigue or loss of energy; PHQ9_6, Worthlessness/Excessive Guilt; PHQ9_8, Psychomotor agitation or retardation; PHQ9_9, Suicidal ideation; PSQI, Pittsburgh Sleep Quality Index; PSQI_1, Sleep quality; PSQI_3, Sleep duration; PSQI_4, Sleep efficiency; PHQ15, Patient, Health Questionnaire-15; PHQ15_7, Dizziness; PHQ15_9, Feeling your heart pound or race; PHQ15_10, Shortness of breath; PHQ15_11, Pain or problems during sexual intercourse; PHQ15_15, Trouble sleeping; FDR, false discovery rate. ^*^p < 0.05 was considered a statistical difference. Note: Only items with statistically significant differences (*p* < 0.05) are displayed. The full list of clinical characteristics is provided in **Supplementary Table 1**. ^a^Mann–Whitney U test. ^b^Chi-square test.

Stratified Analysis of Age and Family History: To validate the robustness of these baseline differences, we conducted stratified analyses (detailed in **Supplementary Tables 2,3**). Age Stratification: Analysis by age quartiles confirmed an age-dependent distribution pattern. The proportion of BD patients was highest in the youngest quartile (12–17 years: 63%) and progressively decreased in the oldest quartile (25–30 years: 27%), reinforcing the association between younger age and BD diagnosis in this sample. Family History Stratification: Further analysis clarified the nature of the family history discrepancy. In the BD group, data quality was the limiting factor. Approximately 67.1% of patients were classified as “Unknown” while only 9.5% had a confirmed positive family history. In the UD group, records were significantly more complete. Only 24.3% were “Unknown” with the majority having confirmed status (48.9% negative and 26.8% positive).

### 3.2 Performance of Classification Models

**Fig. 2.**
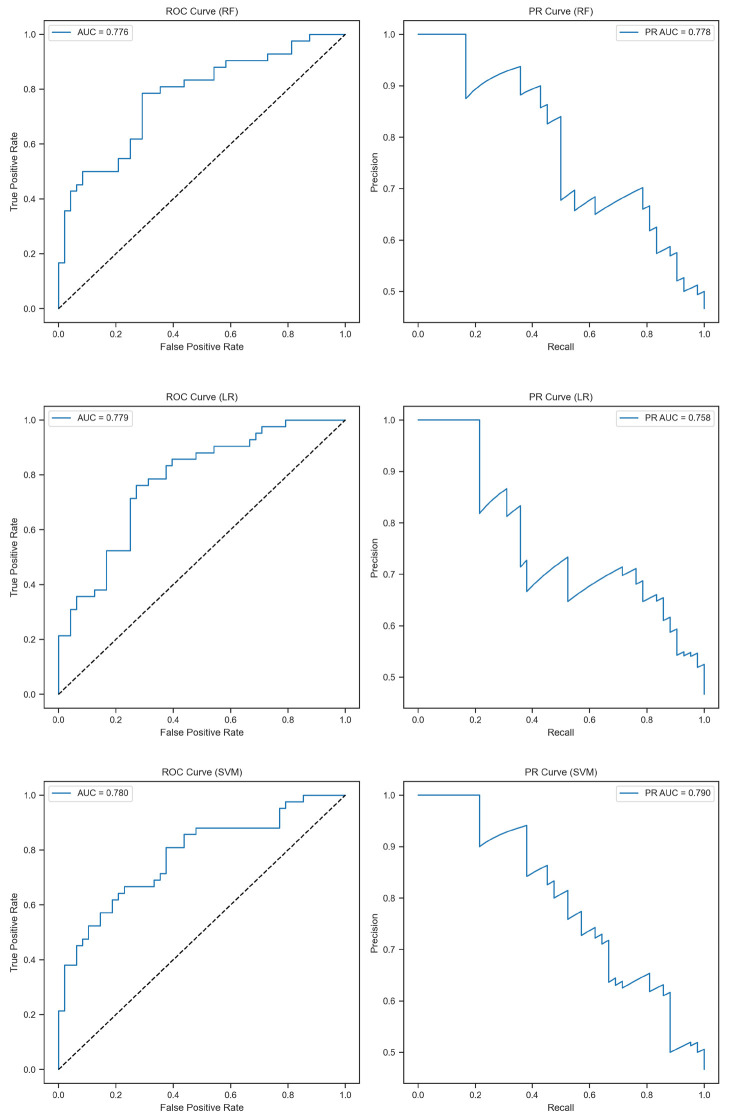
**ROC curves and PR curves for RF, LR, and SVM**. PR, precision-recall; ROC, receiver operating characteristic.

**Fig. 3.**
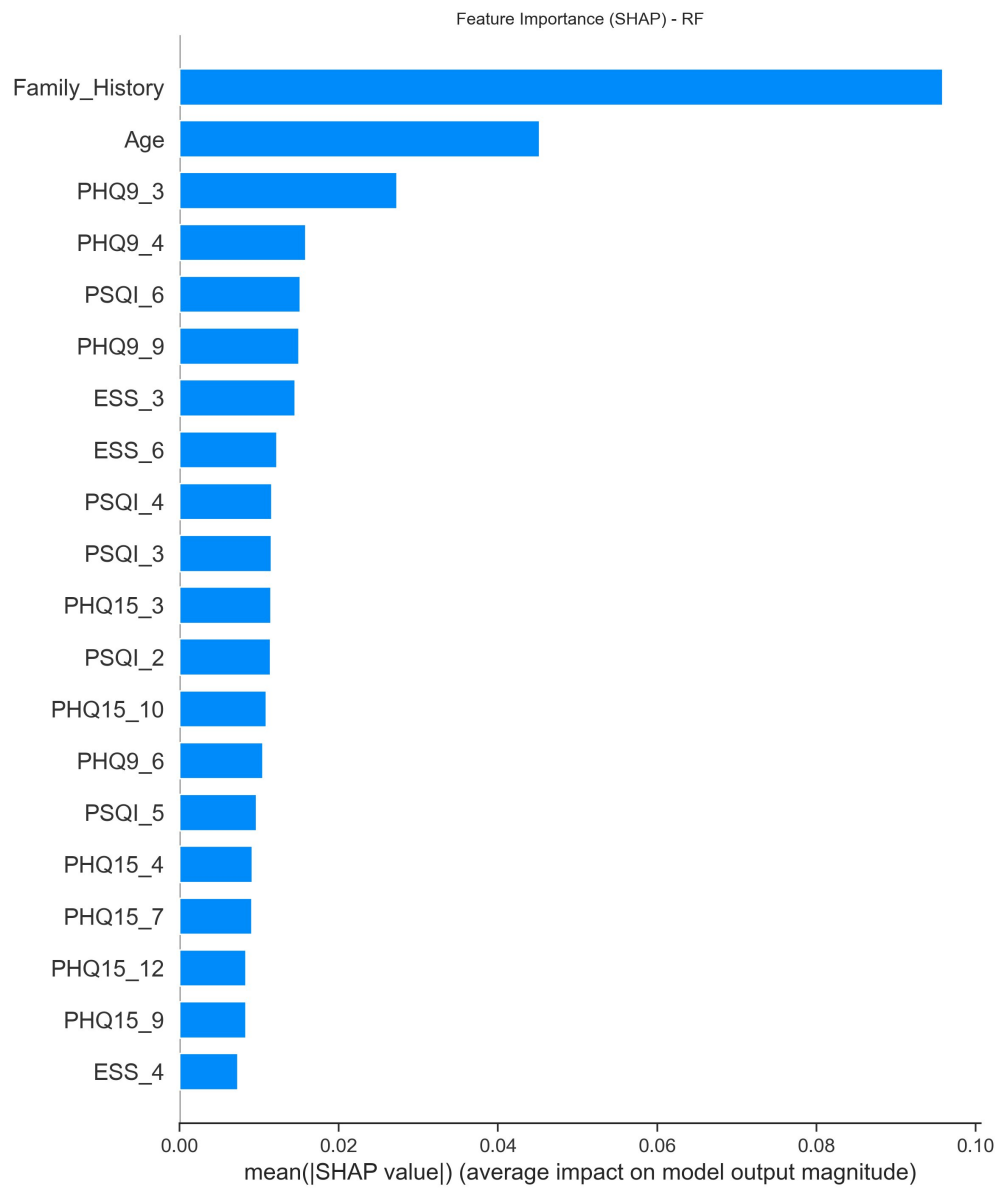
**Feature importance ranking for the Random Forest model based on SHAP values**. ESS, epworth sleepiness scale.

**Fig. 4.**
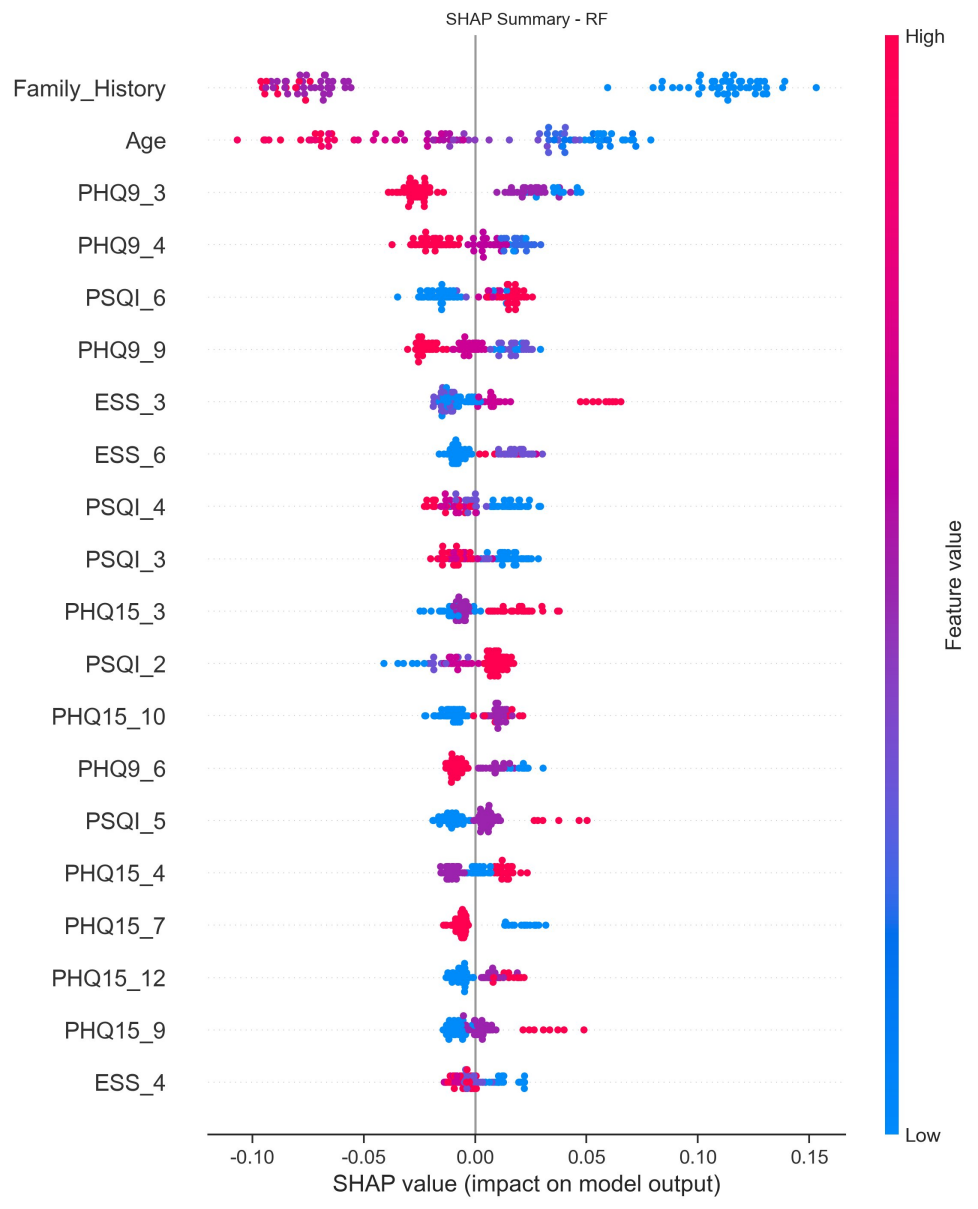
**SHAP values for each feature included in the RF model**. The y-axis ranks the top 20 features by mean influence. The x-axis shows SHAP values, where the left side indicates unipolar depression and the right indicates bipolar depression.

UD from BD using a feature set comprising age, family history of mental disorders, and item-level scores was derived from all clinical scales. Overall performance metrics are summarized in Table 2, and corresponding ROC and precision-recall (PR) curves are shown in Fig. 2; The confusion matrices and calibration curves are detailed in **Supplementary Figs. 1A–C, 2A–C**, respectively. In the nested cross-validation used for model comparison, LR, RF, and SVM showed comparable discrimination (mean AUC ± SD: LR 0.755 ± 0.047; RF 0.747 ± 0.061; SVM 0.739 ± 0.060), with similar calibration error (mean Brier ± SD: LR 0.206 ± 0.013; RF 0.207 ± 0.015; SVM 0.213 ± 0.020). On the independent hold-out test set, ROC analyses indicated broadly similar discrimination. ROC-AUC values clustered around 0.78 (RF: 0.776; LR: 0.779; SVM: 0.780), suggesting a moderate ability to distinguish UD from BD based on the combined demographic and clinical features. Consistent with these findings, overall accuracies and weighted F1 scores differed only modestly (Table 2). However, RF demonstrated a more balanced trade-off between sensitivity and specificity, whereas LR tended to favor higher sensitivity at the cost of lower specificity; SVM performance fell between these two patterns. Notably, inspection of the confusion matrices revealed that RF distributed errors most evenly between the two classes. To ensure reproducibility, the optimal hyperparameters of RF identified via grid search were: n_estimators = 200, max_depth = 10, min_samples_split = 2, and class_weight = None. Given its balanced predictive capability, we proceeded with the RF model for further analysis (Figs. 3,4).

**Table 2.** **Performance metrics for the models**.

Model	Accuracy (95% CI)	AUC (95% CI)	Sensitivity	Specificity	Weighted F1 (95% CI)
RF	0.73 (0.64–0.82)	0.776 (0.68–0.87)	0.76	0.71	0.74 (0.61–0.80)
LR	0.71 (0.61–0.80)	0.779 (0.68–0.86)	0.85	0.58	0.71 (0.60–0.79)
SVM	0.76 (0.57–0.77)	0.780 (0.67–0.86)	0.66	0.68	0.67 (0.57–0.77)

### 3.3 Sensitivity Analysis of Family History Coding

Because the “unknown/uncertain” category could reflect information availability rather than true familial risk, we conducted a sensitivity analysis by re-running a Random Forest model under three strategies: (A) excluding records with unknown family history (N = 250), (B) treating unknown family history as missing and imputing it (N = 449), and (C) modeling unknown family history as its own category (N = 449). Discrimination was highest when unknown was modeled as its own category (AUC = 0.782) and slightly lower when treated as missing (AUC = 0.748), whereas excluding unknown substantially degraded performance (AUC = 0.618) and produced a degenerate sensitivity of 0.0.

### 3.4 Identification of Key Clinical Discriminators for BD and UD via SHAP Analysis

To identify which demographic and clinical features are most critical for distinguishing BD from UD, we calculated SHAP values based on the Random Forest model. SHAP values offer an interpretable method to evaluate how individual features contribute to decision-making. As shown in the feature importance ranking (Fig. 3), family history and age emerged as the dominant predictive factors. Specific clinical scale items—notably PHQ9 items 3, 4, and 9, along with PSQI item 6—were identified as highly influential.

The SHAP summary plot (Fig. 4) further revealed the directional impact of these features. Regarding demographic characteristics, younger age and an “unclear” family history (represented by lower feature values) were strong predictors associated with a diagnosis of BD (positive SHAP values). In terms of symptoms, the use of sleep medication (PSQI item 6) was a significant predictor for BD. In contrast, higher scores on PHQ9 item 9 (suicidal ideation), item 3 (sleep disturbance), and item 4 (fatigue or loss of energy) favored a diagnosis of UD (negative SHAP values).

## 4. Discussion

This study aimed to develop and compare three supervised learning models (RF, LR, and SVM) for distinguishing BD from UD using demographic information and clinical scale profiles. All three models demonstrated comparable discriminative capacity on the test set, with ROC-AUC values around 0.78. This performance falls within the range of established screening instruments like the Mood Disorder Questionnaire(MDQ) and Hypomania Checklist-32 (HCL-32), which generally report AUC values between 0.73 and 0.86 in validation studies and meta-analyses [38]. Given the high rate of initial misdiagnosis (60%) in routine clinical practice [4], our automated approach represents a viable, cost-effective triage tool to identify high-risk individuals warranting specialist assessment, thereby serving as an adjunct to—rather than a substitute for—clinical judgment. Although the AUC values were similar, the RF model exhibited a more balanced error distribution in the confusion matrix and a robust trade-off between sensitivity and specificity. Consequently, RF was selected for interpretability analysis (SHAP) to identify potential clinical discriminators (Figs. 3,4). Clinically, misdiagnosing BD as UD can delay mood stabilizer intervention or induce mania/rapid cycling [39]. Therefore, even a model with moderate AUC holds practical value if it can identify high_contribution features to aid in stratified screening.

In addition, nested cross-validation within the training set showed stable discrimination across folds, supporting that the reported hold-out performance is not driven by a single favorable split. Feature importance analysis of the RF model identified family history and age as key predictors (Fig. 3). Following these were specific depression- and sleep-related items (PHQ9_3, PHQ9_4, PSQI_6, and PHQ9_9). This aligns with existing literature highlighting the significant roles of genetic predisposition and age-related risk patterns in the pathogenesis of BD [40, 41, 42]. These findings comply with the Canadian Network for Mood and Anxiety Treatments (CANMAT) guidelines, which similarly identify sleep changes, rhythm disturbances, and irritability as critical dimensions for differentiating mood disorders [43]. Consistent with prior research, our results underscore that while individual clinical features are informative, they lack sufficient diagnostic specificity in isolation. This necessitates the integration of multidimensional parameters to enhance phenotypic characterization and diagnostic accuracy [44]. From a mechanistic perspective, these distinct symptom profiles likely reflect underlying dynamic network alterations and compensatory neural mechanisms. Recent evidence on naturalistic emotion processing indicates that the fluctuating rhythm and emotional instability characteristic of BD may stem from aberrant dynamic functional connectivity states and a failure in network reorganization during emotional arousal, distinguishing it from the network rigidity often observed in UD [45]. Furthermore, the prominence of reward-related features (e.g., sleep medication use, energy loss) in our model aligns with disrupted thalamo-cortical signaling and reward circuit dissociation, which impairs bottom-up processing and necessitates compensatory neural recruitment to maintain homeostasis [46]. Thus, the demographic and clinical features identified by our machine learning approach may serve as accessible macroscopic proxies for these complex, transdiagnostic neural plasticity and circuit disruption processes [47].

SHAP value analysis provided local explanations for how feature values influenced predictions (Fig. 4). Notably, an “uncertain/unknown” family history (coded as 1) emerged as a significant predictor for BD. This aligns with the significantly higher proportion of “unclear” reports observed in the BD group (**Supplementary Table 3**). It is crucial to interpret this correctly: a low feature value in this context represents “unknown status”, rather than a confirmed “negative history”. Therefore, this finding does not contradict established evidence that a positive family history correlates with high disease risk [48, 49]. Instead, it indicates that the model leveraged this “uncertainty” as a latent marker of the early-onset clinical profile. In clinical practice, obtaining an accurate family history from younger patients with BD can often be challenging [50], due to recall bias, interrupted follow-ups, or stigma-induced concealment. Consequently, “uncertainty” itself becomes a predictive signal. This implies that future standardization for collecting family history could alter the model’s performance and feature rankings, highlighting the necessity for external validation.

To address the concern that this effect could be an artifact of coding or missingness, we conducted a sensitivity analysis under three family history strategies. The results showed that treating “unknown/uncertain” as an explicit category (AUC = 0.782) performed best and was consistent with the main analysis, whereas excluding unknown substantially degraded discrimination (AUC = 0.618) and produced a degenerate sensitivity of 0.0. These findings suggest that the predictive power of the “unknown” category likely stems from the specific demographic or clinical context of these patients (e.g., less contact with caregivers or onset ambiguity) rather than the “unknown” status acting as a biological risk factor itself.

Younger age was associated with a prediction of BD. This is consistent with observations that BD is characterized by earlier onset, a prolonged course, and greater recurrence rates in younger populations [51, 52]. In a hypothetical scenario where age is evenly distributed, the model might lose this critical “early-onset” diagnostic signal. The use of sleep medication (PSQI_6) contributed to predicting BD, whereas suicidal ideation (PHQ9_9), sleep disturbance (PHQ9_3), and fatigue or loss of energy (PHQ9_4) tended to predict UD. This pattern may reflect the fact that BD patients are more frequently treated pharmacologically for sleep rhythm disturbances [53], making the “medication” item a discriminative signal. Conversely, the prominence of specific PHQ9 items in the UD group suggests a more concentrated burden of typical depressive symptoms. Machine learning models excel at capturing these complex, nonlinear relationships, which traditional statistical frameworks may overlook.

Machine learning methodologies have emerged as pivotal tools in the psychiatric domain, offering data-driven insights to refine diagnostic precision, optimize treatment selection, and predict prognostic trajectories [54, 55]. However, translating these algorithms into tangible clinical benefits is impeded by significant conceptual and methodological challenges, notably the “black-box” opacity that often hinders clinical adoption [55] and the risk of overfitting inherent in retrospective, single-site designs [56]. To mitigate these concerns, we implemented a rigorous analytical framework: we employed SHAP analysis to generate interpretable, clinically relevant feature attributions, and utilized nested cross-validation combined with sensitivity analyses to minimize optimism bias and ensure model stability. While external replication remains paramount, these methodological choices represent a critical effort to bridge the gap between computational performance and genuine clinical utility, prioritizing both model transparency and reproducibility.

### Limitations

This study has several limitations. Firstly, the retrospective design and reliance on the “*Good Sleep 365*” mHealth platform imposed specific constraints on data availability and patient selection. Notably, bipolar-specific screening instruments (e.g., HCL-32 or MDQ) were excluded because they were administered selectively based on clinical judgment rather than universally at baseline; including them would have introduced substantial missing data and selection bias. Furthermore, the platform’s mandatory completion requirement likely underrepresented patients with severe psychomotor retardation, acute agitation, or cognitive impairment. Consequently, our findings are best generalized to patients with sufficient functional capacity to engage with digital tools. Secondly, the development and validation of this model were based entirely on a sample aged 12–30 years. Therefore, its performance is primarily applicable within this age range, and caution is warranted when extrapolating to older populations where clinical phenotypes may differ. Relatedly, the relatively small sample size may not fully capture the heterogeneous manifestations of the disorder. Additionally, while excluding common psychiatric comorbidities (e.g., substance use disorders) enhanced internal validity, it limits generalizability to complex real-world settings where comorbidities are prevalent. Third, the cross-sectional nature of the study precludes inferences regarding disease trajectories. Finally, given the prominent contribution of “uncertain” family history, the model’s robustness warrants re-evaluation in scenarios where data collection is more standardized to reduce ambiguity.

Despite these limitations, the model holds significant potential for clinical translation if implemented with appropriate safeguards. To mitigate the risk of antidepressant-induced mania resulting from misdiagnosis, we propose a clinically actionable, sensitivity-optimized threshold strategy (e.g., prioritizing sensitivity >0.85). While LR inherently showed high sensitivity, it suffered from low specificity (high false positives), which could lead to clinical ‘alarm fatigue’. In contrast, the Random Forest model, with its superior balanced error distribution, allows us to tune the threshold for high sensitivity while retaining adequate specificity. In practice, this serves as a decision support tool: for example, in a scenario where a young patient presents with depression and irregular sleep, a positive model flag would not dictate a diagnosis but rather prompt the clinician to screen rigorously for hypomania before prescribing antidepressants.

## 5. Conclusions

This study developed machine learning models using age, family history, and clinical features, achieving moderate efficacy in differentiating UD from BD in adolescent and young adult patients. Among the tested algorithms, the RF model demonstrated a superior balance in error distribution. SHAP analysis indicated that age, family history (specifically the “unknown/uncertain” category), and specific items related to sleep medication and depressive symptoms (e.g., sleep disturbance, fatigue, and suicidal ideation) were significant contributors to the model’s decision-making. Consequently, this model is best positioned as a low-burden screening tool to assist clinical judgment, rather than a replacement for specialist diagnosis. Future research should prioritize multi-center, real-world studies that include patients with common comorbidities. Such studies must incorporate critical data on the bipolar spectrum and disease course, while rigorously controlling for unmeasured confounders and undergoing external validation.

## Data Availability

Due to confidentiality restrictions related to the participants, the data are not publicly available. Data supporting the findings of this study can be obtained from the corresponding author upon reasonable request.

## Abbreviations

Md, median; IQR, interquartile range; GAD7, Generalized Anxiety Disorder 7-item scale; PHQ9, Patient Health Questionnaire-9; PSQI, Pittsburgh Sleep Quality Index; ESS, Epworth Sleepiness Scale; PHQ15, Patient Health Questionnaire-15; UD, unipolar depression; BD, bipolar depression; ROC-AUC, area under the receiver operating characteristic curve; FDR, false discovery rate; ACC, Accuracy.
