# Supplementary material for: Diagnostic Differentiation Between Unipolar and Bipolar Depression: A Machine Learning Analysis of Demographic and Clinical Features
Source: Alpha Psychiatry. 2026 Jun 30;27(3):47274. doi: 10.31083/AP47274 (PMC13339793; doi:10.31083/AP47274)
Supplement: Supplementary file 1 [file 2757-8038-27-3-47274-s1.zip › Supplementary Material.docx]

*Supplementary Materials*

**Diagnostic differentiation between unipolar and bipolar depression: A machine learning analysis of demographic and clinical features**

**(A)**

**
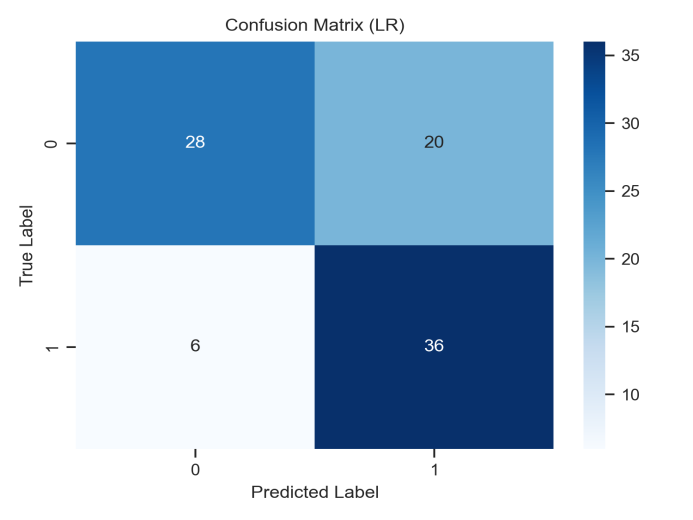
**

**(B)**

**
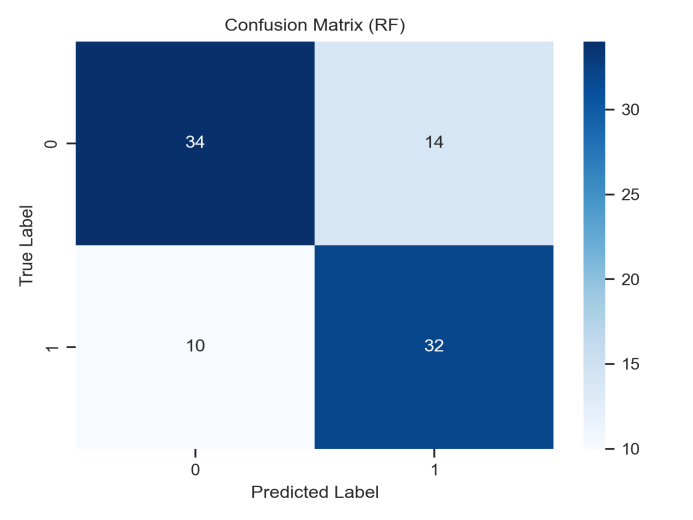
**

**(C)**

**
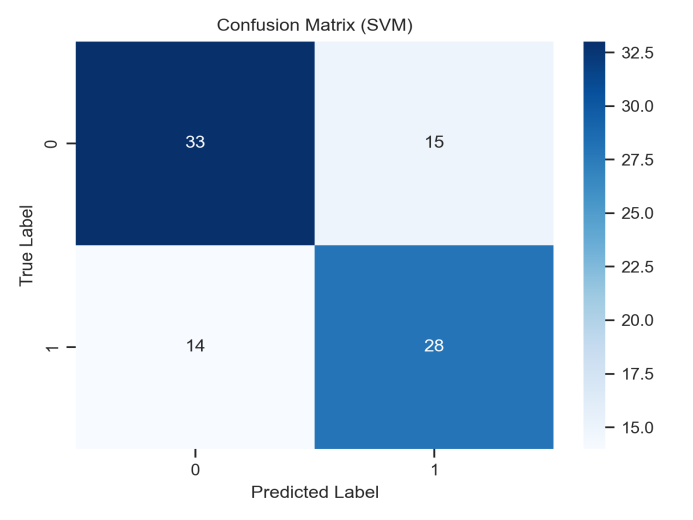
**

**Supplementary Fig. 1 A-C: Confusion Curve of SVM, RF, and LR Model**

**(A)**


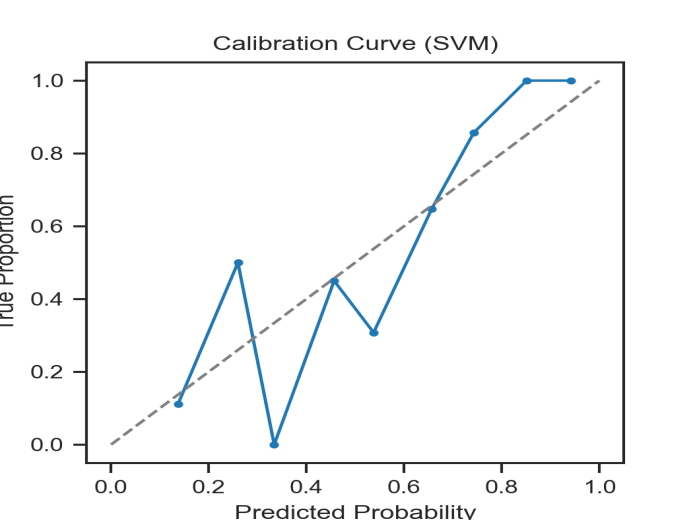


(B)


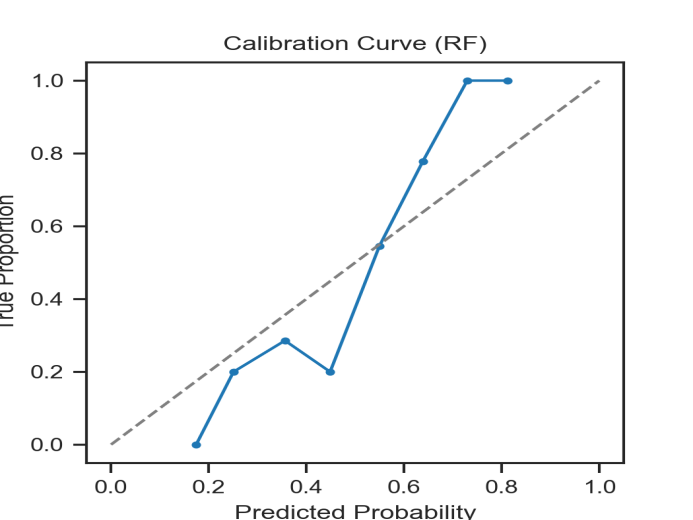


(C)


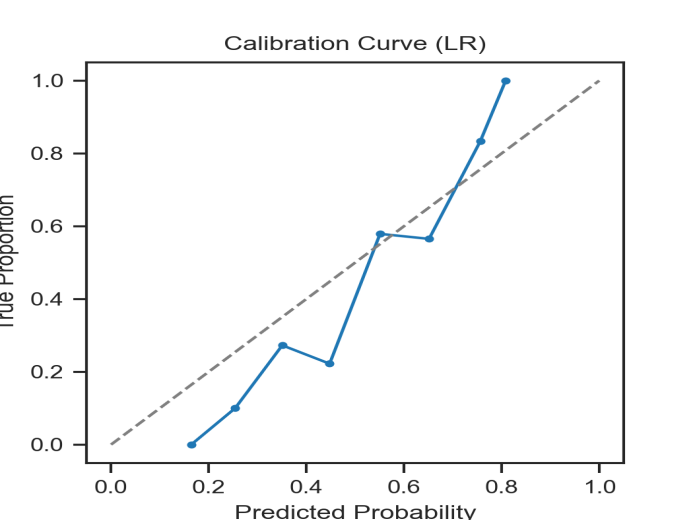


**Supplementary Fig. 2 A-C: Calibration Curve of SVM, RF, and LR Modle**

Supplementary Table 1. Clinical characteristics of UD and BD

| Variables | | UD(239) | BD(210) | χ2 /Z | Effect Size | p | p_(FDR)_ |
| --- | --- | --- | --- | --- | --- | --- | --- |
| Age(years) (Q1,Q3) | | 23 (18,27) | 19(16,23) | -5.7^a^ | -0.31 | <0.001^*^ | <0.001^*^ |
| Sex (female/male) | | 161/78 | 156/54 | 2.6^b^ | 0.07 | 0.120 | 0.30 |
| Educational Level (n,%) | Primary and Secondary School | 89(37.2%) | 109(51.9%) | 9.8^b^ | 0.16 | 0.002^*^ | 0.07 |
|  | University and above | 150(62.8%) | 101(48.1%) |  |  |  |  |
| Family History (N/Y/Unclear)（n,%) | | 117(49.0%)/64(26.8%)/58（24.3%) | 49(23.3%)/20(9.5%)/141(67.1%) | 83.9^b^ | 0.43 | <0.001^*^ | <0.001^*^ |
| GAD7 items Md (Q1,Q3) | GAD7**_**1 | 3(2,3) | 3(2,3) | -0.9^a^ | -0.04 | 0.374 | 0.55 |
|  | GAD7**_**2 | 2 (1,3) | 2 (1,3) | -1.7^a^ | -0.09 | 0.091 | 0.23 |
|  | GAD7**_**3 | 2 (1,3) | 2 (1,3) | -1.7^a^ | -0.09 | 0.098 | 0.23 |
|  | GAD7**_**4 | 2 (1,3) | 2 (1,3) | -2.3^a^ | -0.12 | 0.021* | 0.07 |
|  | GAD7**_**5 | 2 (1,3) | 1(1,2) | -0.2^a^ | -0.01 | 0.807 | 0.92 |
|  | GAD7**_**6 | 2 (1,3) | 2 (1,3) | -0.6^a^ | -0.03 | 0.567 | 0.75 |
|  | GAD7**_**7 | 1(0,2) | 1(1,2) | -0.2^a^ | -0.01 | 0.843 | 0.92 |
| PHQ9 items Md (Q1,Q3) | PHQ9**_**1 | 1(1,2) | 1(1,2) | -0.9^a^ | 0.05 | 0.349 | 0.53 |
|  | PHQ9**_**2 | 1(0,2) | 1(1,2) | -0.4^a^ | 0.02 | 0.674 | 0.78 |
|  | PHQ9**_**3 | 3 (2,3) | 2 (1,3) | -4.0^a^ | 0.20 | <0.001^*^ | <0.001^*^ |
|  | PHQ9**_**4 | 2 (2,3) | 2 (1,3) | -3.7^a^ | 0.19 | <0.001^*^ | 0.02^*^ |
|  | PHQ9**_**5 | 3(2,3) | 3(2,3) | -0.1^a^ | 0.00 | 0.927 | 0.95 |
|  | PHQ9**_**6 | 3 (2,3) | 3 (1,3) | -3.1^a^ | 0.15 | 0.002^*^ | 0.02^*^ |
|  | PHQ9**_**7 | 2 (1,3) | 2 (1,3) | -0.7^a^ | 0.04 | 0.502 | 0.72 |
|  | PHQ9**_**8 | 2 (1,3) | 2 (1,3) | -2.4^a^ | -0.12 | 0.019^*^ | 0.07 |
|  | PHQ9_9 | 2 (1,3) | 2 (1,3) | -2.6^a^ | 0.14 | 0.009^*^ | 0.05 |
| PSQI items Md (Q1,Q3) | PSQI**_**1 | 2 (2,3) | 2 (2,3) | -1.9^a^ | -0.10 | 0.047^*^ | 0.14 |
|  | PSQI**_**2 | 3(2,3) | 3(2,3) | -1.4^a^ | -0.12 | 0.175 | 0.38 |
|  | PSQI**_**3 | 2 (0,3) | 2 (0,3) | -2.9^a^ | -0.15 | 0.004^*^ | 0.03^*^ |
|  | PSQI**_**4 | 2 (0,3) | 1 (0,2) | 2.4^a^ | -0.12 | 0.017^*^ | 0.07 |
|  | PSQI**_**5 | 1(1,2) | 2(1,2) | -1.2^a^ | 0.06 | 0.247 | 0.45 |
|  | PSQI**_**6 | 0(0,3) | 2(0,3) | -1.9^a^ | 0.09 | 0.058 | 0.16 |
|  | PSQI**_**7 | 3(0,0) | 3(3,3) | -1.7^a^ | 0.07 | 0.084 | 0.22 |
| PHQ15 items Md (Q1,Q3) | PHQ15**_**1 | 1(1,2) | 1(1,2) | -0.2^a^ | 0.01 | 0.829 | 0.92 |
|  | PHQ15**_**2 | 0(0,0) | 0(0,0) | -1.1^a^ | -0.03 | 0.267 | 0.46 |
|  | PHQ15**_**3 | 1(0,2) | 1(1,2) | -0.6^a^ | 0.03 | 0.546 | 0.75 |
|  | PHQ15**_**4 | 1(0,2) | 1(0,2) | -0.9^a^ | 0.05 | 0.343 | 0.53 |
|  | PHQ15**_**5 | 1(0,2) | 1(0,2) | -0.4^a^ | 0.02 | 0.653 | 0.78 |
|  | PHQ15**_**6 | 1(0,2) | 1(1,2) | -0.6^a^ | 0.03 | 0.567 | 0.75 |
|  | PHQ15**_**7 | 2 (2,2) | 2 (1,2) | -3.1^a^ | -0.12 | 0.002^*^ | 0.02^*^ |
|  | PHQ15**_**8 | 2(1,2) | 2(1,2) | -1.2^a^ | -0.05 | 0.219 | 0.42 |
|  | PHQ15**_**9 | 0 (0,1) | 1 (0,1) | -2.6^a^ | 0.13 | 0.009^*^ | 0.05 |
|  | PHQ15**_**10 | 0 (0,1) | 1 (0,1) | -1.9^a^ | 0.10 | 0.049^*^ | 0.14 |
|  | PHQ15**_**11 | 0 (0,1) | 1 (0,1) | -2.1^a^ | 0.10 | 0.038^*^ | 0.13 |
|  | PHQ15**_**12 | 0 (0,1) | 0 (0,1) | -1.0^a^ | 0.05 | 0.310 | 0.50 |
|  | PHQ15**_**13 | 0 (0,0) | 0 (0,0) | -0.4^a^ | -0.02 | 0.654 | 0.78 |
|  | PHQ15**_**14 | 1 (1,1) | 1(0,1) | -0.1^a^ | -0.00 | 0.920 | 0.95 |
|  | PHQ15**_**15 | 0 (0,1) | 0 (0,1) | -2.4^a^ | 0.11 | 0.017^*^ | 0.07 |

Md:median, Q1:25th percentile, Q3:75th percentile, GAD7:Generalized Anxiety Disorder 7-item scale, PHQ9:Patient Health Questionnaire-9, PSQI :Pittsburgh Sleep Quality Index, PHQ15:Patient:Health Questionnaire-15, UD:Unipolar Depression, BD:Bipolar Depression, *p < 0.05 was considered a statistical difference. ^a^ Mann-Whitney U test. ^b^ Chi-square test.

**Stratified Analysis of Age and Family History:**

We stratified the entire sample into four groups based on age quartiles and calculated the proportion of BD and UD within each group(Table S2). This allowed us to observe potential systematic shifts in diagnostic distribution across age levels and verify the robustness of the conclusion that BD is associated with younger age.

**Supplementary Table 2 Stratified Analysis of Age**

| **age_bin** | **BD** | **UD** |
| --- | --- | --- |
| (12,17) | 63% | 38% |
| (17.,21) | 51% | 49% |
| (21,25) | 44% | 56% |
| (25,30) | 27% | 73% |

**Methodology & Descriptive Results:**

The 'Family History' variable was treated as a ternary variable with three distinct levels: -1 (unknown/unrecorded), 0 (no family history), and 1 (positive family history). Proportions of BD and UD were calculated within each stratum (Table S3). Within the BD group, the majority (67.1%) were classified as 'unknown' (-1), while 23.3% had no family history and 9.5% had a positive family history. In contrast, within the UD group, 24.3% were classified as 'unknown', only had no family history 48.9% and 26.8% had a positive family history .

**Supplementary Table 3 Stratified Analysis of Family History**

| **Diagnosis** | **prop** | **Mean(years)** | **std** | **50%** | **family_history** |
| --- | --- | --- | --- | --- | --- |
| BD | 0.71 | 19.39 | 4.59 | 18 | -1 |
| UD | 0.29 | 22.40 | 4.87 | 22.5 | -1 |
| BD | 0.30 | 20.98 | 4.84 | 20 | 0 |
| UD | 0.70 | 22.38 | 4.99 | 22 | 0 |
| BD | 0.24 | 20.80 | 3.14 | 20 | 1 |
| UD | 0.76 | 22.86 | 4.24 | 23 | 1 |
